# Research Trends and Knowledge Atlas of Radiotherapy-Related Cognitive Impairment: A Bibliometric Analysis

**DOI:** 10.2174/011570159X368986250415105149

**Published:** 2025-05-09

**Authors:** Yaqian Tan, Qi Song, Siyuan Gao

**Affiliations:** 1 Department of Pharmacy, The Affiliated Brain Hospital, Guangzhou Medical University, Guangzhou, China;; 2 Key Laboratory of Neurogenetics and Channelopathies of Guangdong Province and The Ministry of Education of China, Guangzhou Medical University, Guangzhou, China;; 3 Department of Pharmacy, Guangzhou Institute of Cancer Research, The Affiliated Cancer Hospital, Guangzhou Medical University, Guangzhou, China;; 4 Department of Pharmacy, Guangdong Province Hospital for Occupational Disease Prevention and Treatment, Guangzhou, China

**Keywords:** Radiotherapy, radiation, cognitive impairment, visualization, bibliometric analysis, VOSviewer, R-*bibliometrix*

## Abstract

**Background:**

Radiotherapy is one of the main therapeutic methods for tumors, and radiation-related cognitive impairment has attracted increasing attention. The purpose of this study was to explore the research prospects in the field of radiotherapy-associated cognitive decline through bibliometric analysis.

**Methods:**

Literature on radiotherapy-related cognitive impairment published during 2004-2023 were extracted from the Web of Science Core Collection database. VOSviewer and R-*bibliometrix* were utilized to perform bibliometric analysis.

**Results:**

A total of 8,365 publications were retrieved from the database. The United States emerged as the leading country in this research field, with St. Jude Children's Research Hospital identified as the most productive institution. Thomas E. Merchant was the most prolific author in this field, while Charles L. Limoli was the most frequently cited scholar. The research hotspots have gradually shifted from cognitive function and outcome measurement to the development of new therapy models.

**Conclusion:**

This study comprehensively examined the research hotspots and knowledge atlas of radiotherapy-related cognitive decline from a bibliometric perspective. Our results would assist scholars in identifying potential collaborators and significant literature in this field while also providing valuable guidance for future research directions.

## 1 INTRODUCTION

Radiotherapy is one of the main therapeutic methods for tumors, along with surgical resection and systemic therapy [1]. While radiotherapy has definite survival benefits for many cancers, healthy tissues surrounding tumors can also be damaged by radiation [1, 2]. Evidence suggests that 50-90% of patients encounter progressive and disabling cognitive dysfunctions after radiation exposure [3-6]. These symptoms often include deficits in attention, processing speed, and memory, which severely affect the quality of life for patients [7-9].

Despite the growing focus on radiation-related cognitive impairment, the mechanisms underpinning this syndrome remain largely elusive [10]. There have been reviews summarizing the knowledge of neurocognitive disorders following radiation treatments [4, 11, 12]. However, despite the rapid development and growing body of research on this topic, it remains essential for readers to identify the key research hotspots and determine suitable directions from the vast amount of literature available. Hence, it would be valuable to conduct an analytical investigation of this research area at a macro level.

The approach of bibliometrics applies mathematical and statistical methods [13] and allows researchers to visualize the knowledge atlas of massive data through quantitative analysis and trajectory tracing [14-16]. The co-occurrence networks can reveal the evolutionary process of a topic, and co-authorship patterns can offer potential opportunities for interdisciplinary collaborations [17]. Thus far, no study has analyzed the literature in the field of radiotherapy-related cognitive impairment from a bibliometric perspective. To address this gap, we, therefore, conducted an inclusive bibliometric analysis of existing publications over the last two decades, aiming to provide in-depth insights into the knowledge framework and research frontiers of this field.

## 2 MATERIALS AND METHODS

**Fig. (1):**
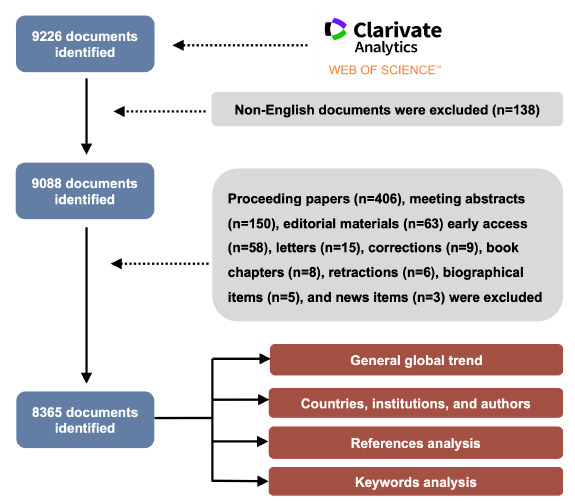
Flowchart of data collection process.

Documents were retrieved from the Web of Science Core Collection database on July 24, 2024. The inclusion criteria was as follows: literatures published during 2004-2023 and were extracted using the search string of “TS = (cognitive* OR neurocognitive*) AND TS = (radiation OR irradiation OR radiotherapy OR radio therapy)”. A total of 9,088 documents were identified after excluding non-English publications. Subsequently, document types, including proceeding papers, meeting abstracts, editorial materials, early access, letters, corrections, book chapters, retractions, biographical items, and news items, were omitted. Ultimately, we identified a total of 8,365 publications for further analysis (Fig. **1**).

In this study, VOSviewer (version 1.6.20) and R-*bibliometrix* (version 4.1.0) were applied for data mining and visualization. VOSviewer is a software under the JAVA environment with the ability to process and present massive bibliometric data [18-22], whereas the R package *bibliometrix* is able to systematically analyze bibliometric sources [21-25]. In this study, we used VOSviewer to construct co-authorship and co-occurrence maps. The data extraction of outputs, collaborations, sources, and keywords was conducted by R-*bibliometrix*.

## 3 RESULTS

### 3.1 Analysis of Global Trends

A total of 8,365 documents were identified after data screening procedures. As illustrated in Fig. (**2**), the annual global production from 2004 to 2023 reflected a constantly ascending tendency, with an average yearly growth rate of 10.84%. The amount of global output peaked in 2022 with 871 publications, whereas the number of total citations reached the highest in 2023 at 30,643.

A total of 115 countries/regions were identified in this study. As displayed in Fig. (**3A**), the USA (n=2,928) was the largest contributor, followed by China (n=1,521) and Germany (n=568). Fig. (**3B**) shows that the top three countries in total citations were the USA (n=90,384), China (n=24,481), and the UK (n=11,185). Fig. (**3C**) illustrates the global distribution map of the top twenty countries in publication. Fig. (**3D**) displays the temporal visualization for country co-authorship networks. The size and color of the node reflected the academic output and activity of a country, while the strength of collaborations between countries was indicated by the thickness of the connecting line.

### 3.2 Analysis of Institutions and Authors

There were 7,200 institutions that participated in the research of this field. St. Jude Children's Research Hospital was the most fruitful institution (n=361), followed by the University of California San Francisco (n=331) and the University of Toronto (n=244) (Table **1**). The temporal evolution of institutional output is represented in Fig. (**4A**). The node size represented the output of an institution, while the color reflected the chronological trend of academic activity. The institutional co-authorship networks in citations are presented in Fig. (**4B**), and the size of the node indicates the total citations of an institution according to the scale.

In this study, we found 32,073 authors devoted themselves to the research of radiotherapy-associated cognitive dysfunction. Thomas E. Merchant was the most productive author (n=57), followed by Charles L. Limoli (n=54), Jing Li (n=51), and Jacob Raber (n=51) (Table **2**). On the other hand, the top three most cited authors were Charles L. Limoli (n=1,214), Minesh P. Mehta (n=872), and John R. Fike (n=866) (Table **2**). The collaborative clusters among authors are displayed in Fig. (**5A**), and the size of the node suggests the scientific production of an author. Fig. (**5B**) represents the chronological pattern of co-authorship networks, and the color scale indicates the active period of an author.

### 3.3 Analysis of Journals and References

The impact factor and journal citation reports are common indicators applied to measure the academic impact of journals [26]. In our present study, we detected 2,028 journals and 338,658 citations in total. Table **3** lists the leading journals in publications, and the top three were IEEE Transactions on Nuclear Science (n=159), Journal of Neuro-Oncology (n=138), and International Journal of Radiation Oncology Biology Physics (n=122). According to the Journal Citation Reports 2023, eight of the top ten most productive journals were distributed in the Q1 region. Neuro*-*Oncology ranked first, with the highest impact factor of 16.4 .As shown in Table **4**, the Journal of Clinical Oncology (n=9,096) had the highest citation, followed by the International Journal of Radiation Oncology Biology Physics (n=7,540) and IEEE Transactions on Nuclear Science (n=4,836). Eight of the top ten most cited journals were Q1 journals, and Nature had the greatest impact factor of 50.5. These data revealed the high quality of the outputs, and would help researchers finding the core journals in this field.

In bibliometrics, there are two indexes assessing the academic influence of the reference, namely “local citation” and “global citation”, which represent the academic impact in similar fields and across fields, respectively [16, 27]. In this study, we extracted a total of 259,800 relevant references. The publication in *Journal of Clinical Oncology* by Vinai Gondi *et al.* had the highest local citation of 208 (Table **5**), while the manuscript published in *Lancet Oncology* by Eric L. Chang *et al.* had the greatest global citation of 1,873 (Table **6**).

### 3.4 Analysis of Keywords and Hotspots

In this study, a total of 14,080 keywords were collected and grouped into four co-occurrence clusters. As demonstrated in Fig. (**6A**), the red cluster included keywords of “memory”, “brain”, “cognition”, “cognitive impairment”, “hippocampus”, and “neurogenesis”. The blue cluster included “radiation”, “impact”, “algorithm,” “model,” and “deep learning.” The green cluster mainly contained “radiotherapy”, “cognitive function”, “quality-of-life”, “cancer”, “surgery”, and “survival”. In the yellow cluster, “children”, “brain tumors”, “long-term survivors”, and “working memory” were the main nodes included. In Fig. (**6B**) exhibiting the chronological evolution of keywords, we found that the earlier hotspots included “quality of life”, “cognitive function”, “survivors”, and “fatigue”, and gradually shifted to “stereotactic radiosurgery”, “management”, “model”, and “immunotherapy”. This suggested that the theme has been merged into clinical practice with other frontier topics, including outcome measurement for patients, risk management, and the development of new therapy models.

## 4 DISCUSSION

### 4.1 General Trends

Annual production and citation are considered intuitive indicators of the development of a research domain [28, 29]. In this study, a significant rising trend in the number of publications and citations was observed, suggesting an increasing demand for effective treatment of radiotherapy-related cognitive decline. Such a trend can be attributed to a variety of factors, including the advancement of radiotherapy technology, prolonged survival time of tumor patients, and the increasing awareness of adverse effects of radiotherapy. As an interdisciplinary subject, the development of this field also benefited from the rapid advancement in neurology, immunology, and oncology fields [30-32]. In addition, our results showed that the USA possessed most of the productive institutions and prolific researchers in the area, reflecting the deep academic accumulation and dominance of this field. Taken together, we envision that this research domain would receive continuous attention, and the USA would maintain its leading position in this field.

### 4.2 Research Hotspots

#### 4.2.1 Mechanisms of Radiation-related Cognitive Decline

The mechanisms underlying radiation-associated cognitive decline have been reported to involve multiple hypotheses, including reduced hippocampal neurogenesis, vascular damage, and neuroinflammation [33]. In the 1970s, findings from rodent models suggested that hippocampal neurons showed altered firing patterns and reduced synaptic efficacy after 4-5 Gy of irradiation, indicating an early phase hippocampal damage induced by radiation [34, 35]. Subsequent studies demonstrated that irradiation-related hippocampal impairment was caused by differentiated neural cell injury and reduced hippocampal plasticity [36, 37]. According to this theory, recent evidence has suggested that human neural stem cell transplantation in mice can alleviate radiation-induced cognitive dysfunction [38]. Findings from rodent preclinical studies and human neuroimaging research have consistently shown that neural progenitor cells in the subgranular zone of the dentate gyrus are highly radiosensitive [39]. Subgranular zone neurons are strongly associated with hippocampal plasticity and cognitive function, and irradiation induces decreased neurogenesis in the subgranular zone [40].

Evidence from both animal and human patients suggested that radiation may cause cognitive dysfunction through vascularization rarefaction. Early in the 1950s, vascular disruption following radiation was first reported in rat models [41], and findings from animal models further suggested that radiation can cause rapid destabilization of the vascular endothelium [42]. Likewise, findings from human studies have shown that adverse reactions caused by endothelial degeneration can manifest months to years following radiation, including intracerebral microvascular dilatation, vascular wall thickening, and small vessel hyalinization [33]. Such vascular damage can lead to decreased cerebral blood flow, ischemic stroke, brain tissue necrosis, and cognitive decline [43]. With the advances in liquid biopsy technologies, we suggest that translational research should focus on hematological biomarkers that are closely correlated with cognitive function and vascular injury, such as serum interleukin-2 and interleukin-6 [44, 45]. These new directions would facilitate the early detection of radiotherapy-induced cognitive impairment.

As for the mechanisms of neuroinflammation, astrocytes, microglia, and neurons have long been thought to be closely associated with radiation-related cognitive impairment [46]. Radiation induces widespread neuroinflammation *via* the activation of astrocytes and microglia, which affects the white matter integrity and brain function [47]. Besides, neuroinflammation can also inhibit neurogenesis, causing inflammation in the hippocampal microenvironment and microvascular damage [48]. Inflammatory markers, such as interleukin, tumor necrosis factor, nuclear factor kappa-B, and glial fibrillary acidic protein, have been identified in irradiated brain tissue [49]. Therefore, it would be instructive to pay more attention to these indicators during and after radiotherapy.

#### 4.2.2 Measures of Radiotherapy-related Cognitive Impairment

With the continuous advancement of radiotherapy technologies, novel methods, such as intensity-modulated radiotherapy, stereotactic radiosurgery, and hippocampal avoidance-whole brain radiotherapy, have been reported to inhibit brain tissue damage and cognitive decline [50-52]. In addition, the use of low radiation doses was recommended, yet its effects on cognition remain debatable [53]. Some evidence suggested that memory impairment was associated with low radiation doses [54], while other studies showed that high doses of direct radiation to the brain caused cognitive impairment in patients [55, 56].

In terms of pharmacological measures, a limited understanding of the mechanisms underlying radiation-related cognitive impairment has driven researchers to select therapeutic candidates from other central nervous system disorders. One strategy is the use of neuroprotective drugs, including memantine, methylphenidate, and donepezil [57]. Results from clinical trials showed that memantine delayed the onset time of cognitive disability and was well-tolerated [58]. The acetylcholinesterase inhibitor donepezil also showed significant improvement in cognitive function after brain irradiation [59]. Similarly, methylphenidate demonstrated positive outcomes of cognitive protection in patients with brain tumors receiving brain radiotherapy [60].

The inhibition of renin-angiotensin system-mediated neuroinflammation can regulate radiation-induced cognitive deficits [61]. A previous rat study found that post-irradiation administration of angiotensin-converting enzyme inhibitor ramipril reduced apoptosis of subgranular zone progenitors [62]. In addition, peroxisomal proliferator-activated receptors (PPARs) are thought to produce anti-inflammatory and neuroprotective effects [63]. Findings in mice showed that administration of PPARα agonist fenofibrate after whole-brain radiation inhibited neuroinflammation and protected hippocampal neurons [64].

It is worth noting that stem cell therapy might have the potential to address radiotherapy-related neurocognitive disorders. There has been evidence that human neural stem cell transplantation alleviated cognitive impairment in irradiated mice [65]. Findings from mice studies further revealed that intranasal delivery of human mesenchymal stem cells promoted recovery from radiation-induced brain injury [66]. In ongoing clinical trials, the employment of intravenous stem cell administration showed much less invasiveness compared to intracranial injection, indicating promising prospects for future treatments in this field [67].

Exercise is an important non-pharmacological option to improve the quality of life for patients. Preclinical studies using mice discovered that animals exhibited better spatial memory recall and hippocampal neuron recovery after physical training of voluntary running [68]. Another study further showed that exercise can ameliorate radiation-related cognitive decline by increasing intracerebral blood oxygen levels and regulating neurotransmission systems [69].

### 4.3 Outlook

Radiotherapy-related cognitive impairment has been recognized as a complex issue involving many factors, such as tumor progression, depression, anxiety, fatigue, sleep dysfunction, and pain [70]. Moreover, anti-cancer therapies, such as chemotherapy, immunotherapy, hormonal therapy, and anti-angiogenic therapy, may also carry the risk of developing cognitive disorders [71, 72]. Notably, the characteristics of radiation-related cognitive impairment vary depending on cancer types. For example, radiotherapy in head and neck cancers causes damage mainly to the temporal lobes, brain stem, and hippocampus, resulting in hearing toxicity, memory deficits, and hypothyroidism [73, 74]. Whereas for primary brain tumors or brain metastases, the most commonly affected brain structures are the hippocampus, prefrontal cortex, and frontal-subcortical network [75, 76]. In this context, the most affected neurocognitive aspects are learning and memory, executive function, and information processing [77]. Furthermore, the cognitive outcomes and recovery rates vary between different time points after radiotherapy [78]. Cognitive impairment occurs 6 months after radiation is believed to be progressive and irreversible [51], with early symptoms manifested as deficits in working memory, attention, and processing speed [79, 80]. Therefore, it would be important for healthcare staff to raise their awareness on this point.

Although encouraging treatments against radiation-associated cognitive decline have been developed, most studies were conducted in rodent models. The significant drawback of animal models is the lack of anatomical and physiological similarity to humans, which limits the translation of findings to humans [81, 82]. Therefore, future investigations should properly combine pre-clinical and clinical work to reveal the translational potential. Moreover, recent developments in brain imaging, including fiber tracking and functional magnetic resonance imaging, would be beneficial for monitoring brain microstructural changes [83] and allow for precise dose constraint and brain damage minimization [84]. To aggregate, these elements would contribute to the early intervention for patients and gain in-depth understanding of this field on multiple levels.

## 5 LIMITATIONS

This study should be interpreted within its limitations. First, the language of publications was restricted to English, and insights from studies written in other languages were neglected. Second, the published papers were restricted to the Web of Science Core Collection database, which might overlook relevant literature from other sources, including Scopus, PubMed, Microsoft Academic, and Google Scholar. At last, although the data were well standardized, bias could still exist due to the presence of various expressions for the same author names or keywords.

## CONCLUSION

In summary, this study investigated the knowledge atlas of literature on radiotherapy-related cognitive impairment from the view of bibliometrics. Firstly, the research in this domain has developed rapidly and is still in its growth stage. The USA possessed the leading position in academic output and distribution. Secondly, prolific authors, institutions, and outstanding journals with highly cited publications have greatly advanced this field. Thirdly, the research frontiers have gradually shifted from cognitive function and outcome measurement to the development of new therapy models. The changing pattern of research hotspots reflected a potential theoretical basis for future research. Our results provided more understanding and guidance for the future directions of this research topic.

## AUTHORS’ CONTRIBUTIONS

YT proposed the topic. YT, QS, and SG collected the data and generated the figures. YT and QS analyzed the data. YT wrote the manuscript. All authors contributed to the manuscript revision and approved the submitted version.

## Figures and Tables

**Fig. (2):**
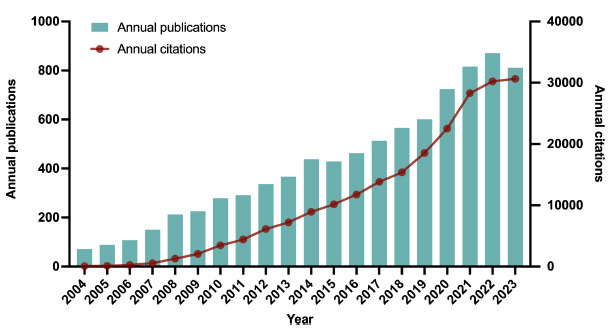
Global trends of publication and citation during 2004-2023.

**Fig. (3):**
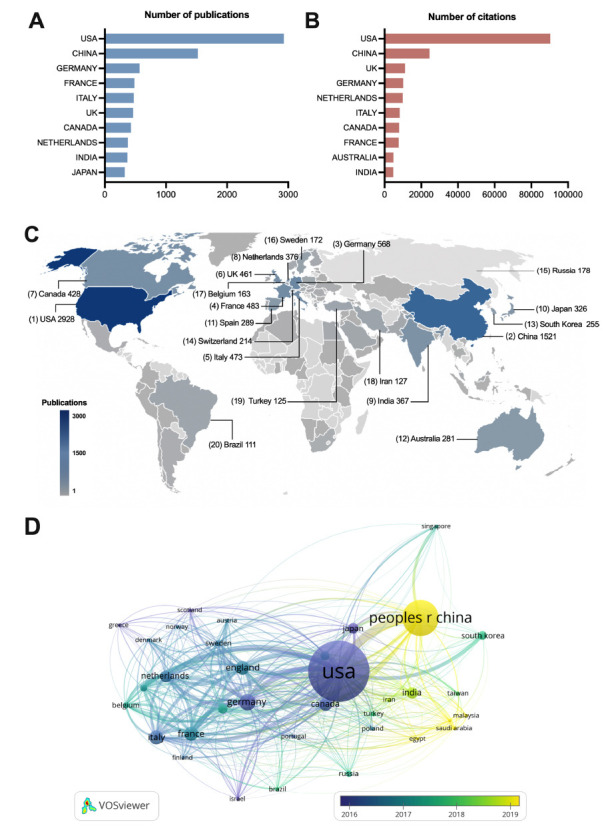
Networks of global contributions. (**A**) The top ten most productive countries; (**B**) The top ten most cited countries; (**C**) Distribution map of the top twenty countries in publication; (**D**) Temporal evolution of country’s co-authorship.

**Fig. (4):**
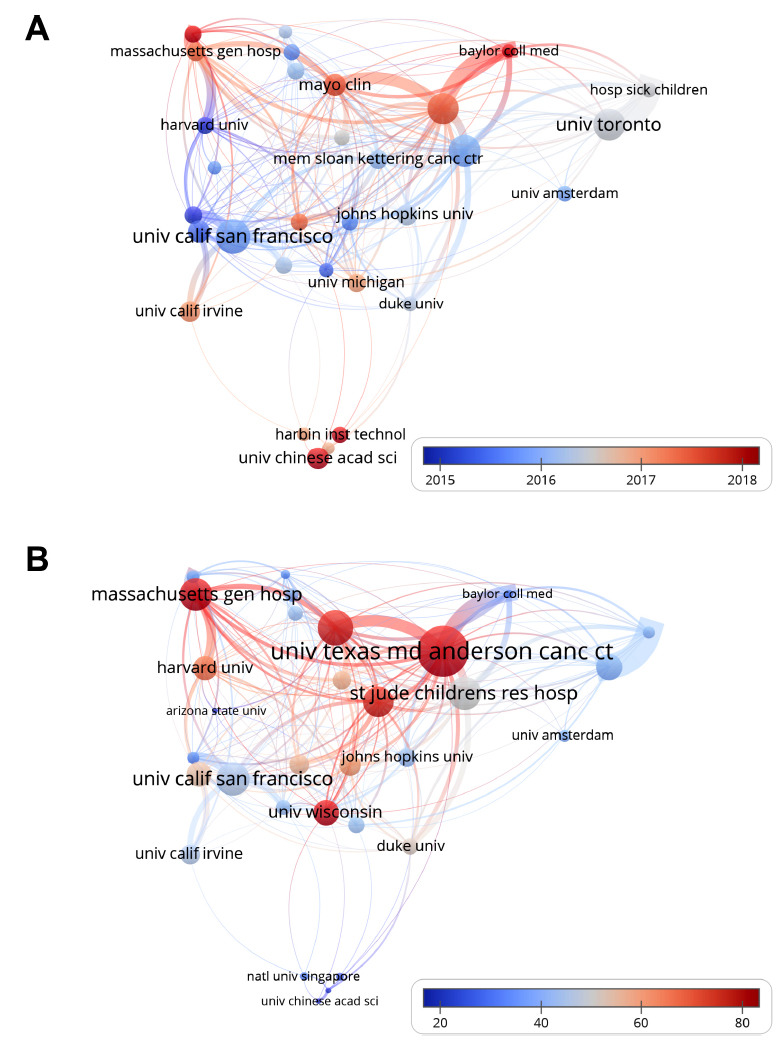
Institutional co-authorship networks of (**A**) temporal evolution and (**B**) citation.

**Fig. (5):**
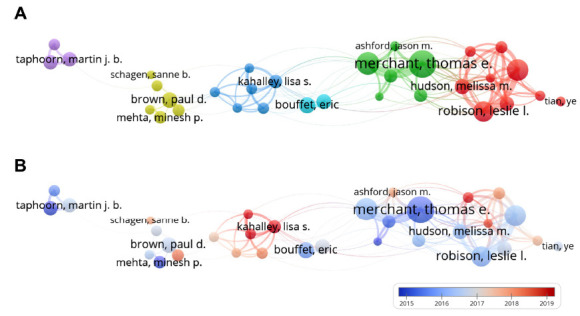
Author co-authorship networks of (**A**) clusters and (**B**) temporal evolution.

**Fig. (6):**
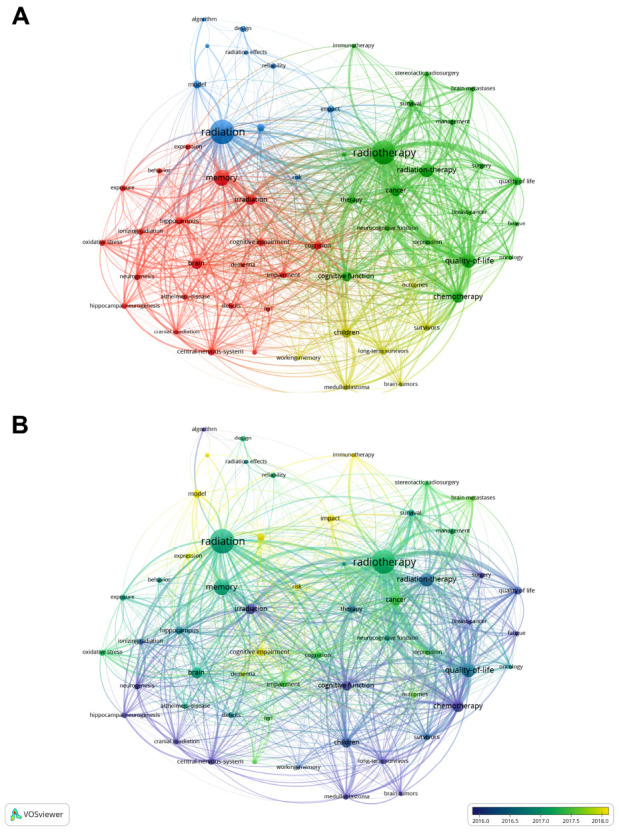
Keywords co-occurrence networks of (**A**) clusters and (**B**) temporal evolution.

**Table 1:** The top ten most productive institutions.

**Rank**	**Institution**	**Country**	**Publications**
1	St. Jude Children's Research Hospital	USA	361
2	University of California San Francisco	USA	313
3	University of Toronto	Canada	244
4	University of Texas MD Anderson Cancer Center	USA	201
5	Oregon Health and Science University	USA	192
6	Johns Hopkins University	USA	166
7	University of Michigan	USA	163
8	University of California San Diego	USA	160
9	Mayo Clinic	USA	141
10	Stanford University	USA	137

**Table 2:** The top ten most prolific authors and the top ten most cited authors.

**Rank**	**Author**	**Publications**	**Rank**	**Author**	**Total citations**
1	Merchant, Thomas E.	57	1	Limoli, Charles L.	1,214
2	Limoli, Charles L.	54	2	Mehta, Minesh P.	872
3	Li, J.	51	3	Fike, John R.	866
4	Raber, Jacob	51	4	Acharya, Munjal M.	805
5	Conklin, Heather M.	46	5	Rosi, Susanna	798
6	Zhang, Y.	45	6	Raber, Jacob	749
7	Krull, Kevin R.	42	7	Robbins, Mike E.	728
8	Wang, J.	40	8	Brown, Paul D.	713
9	Robison, Leslie.	39	9	Parihar, Vipan K	689
10	Wang, H.	38	10	Merchant, Thomas E.	684

**Table 3:** The top ten most fruitful journals.

**Rank**	**Journal**	**Publications**	**Impact Factor (2023)**	**Region**
1	*IEEE Transactions on Nuclear Science*	159	1.9	Q1
2	*Journal of Neuro-Oncology*	138	3.2	Q2
3	*International Journal of Radiation Oncology Biology Physics*	122	6.4	Q1
4	*Radiation Research*	115	2.5	Q2
5	*Scientific Reports*	94	3.8	Q1
6	*PLoS One*	93	2.9	Q1
7	*Neuro-Oncology*	90	16.4	Q1
8	*Physical Review D*	88	5.0	Q1
9	*IEEE Transactions on Antennas and Propagation*	81	4.6	Q1
10	*Cancers*	64	4.5	Q1

**Table 4:** The top ten most cited journals.

**Rank**	**Journal**	**Total Citations**	**Impact Factor (2023)**	**Region**
1	*Journal of Clinical Oncology*	9,096	42.1	Q1
2	*International Journal of Radiation Oncology Biology Physics*	7,540	6.4	Q1
3	*IEEE Transactions on Nuclear Science*	4,836	1.9	Q1
4	*Radiation Research*	4,762	2.5	Q2
5	*Neuroimage*	4,053	4.7	Q1
6	*Nature*	3,262	50.5	Q1
7	*PNAS*	3,107	9.4	Q1
8	*PLoS One*	3,083	2.9	Q1
9	*Journal of Neuro-Oncology*	3,023	3.2	Q2
10	*Physical Review D*	2,963	5.0	Q1

**Table 5:** The top ten local-cited references.

**Rank**	**Reference**	**DOI**	**Local Citations**
1	Gondi V, 2014, *J CLIN ONCOL*	10.1200/JCO.2014.57.2909	208
2	Chang EL, 2009,* LANCET ONCOL*	10.1016/S1470-2045(09)70263-3	201
3	Douw L, 2009, *LANCET NEUROL*	10.1016/S1474-4422(09)70204-2	168
4	Brown PD, 2013, *NEURO-ONCOLOGY*	10.1093/neuonc/not114	146
5	Makale MT, 2017, *NAT REV NEUROL*	10.1038/nrneurol.2016.185	135
6	Greene-schloesser D, 2013, *CLIN CANCER RES*	10.1158/1078-0432.CCR-11-2903	119
7	Meyers CA, 2006, *J CLIN ONCOL*	10.1200/JCO.2005.04.6086	117
8	Brown PD, 2016, *JAMA-J AM MED ASSOC*	10.1001/jama.2016.9839	117
9	Gondi V, 2010, *RADIOTHER ONCOL*	10.1016/j.radonc.2010.09.013	103
10	Brown PD, 2020, *J CLIN ONCOL*	10.1200/JCO.19.02767	101

**Table 6:** The top ten global-cited references.

**Rank**	**Reference**	**DOI**	**Global Citations**
1	Chang EL, 2009, *LANCET ONCOL*	10.1016/S1470-2045(09)70263-3	1,873
2	Brown PD, 2016, *JAMA-J AM MED ASSOC*	10.1001/jama.2016.9839	1,130
3	Snyder JS, 2011, *NATURE*	10.1038/nature10287	1,080
4	Todorov IT, 2006, *J MATER CHEM*	10.1039/b517931a	947
5	Dovedi SJ, 2014,* CANCER RES*	10.1158/0008-5472.CAN-14-1258	925
6	Kloxin CJ, 2013, *CHEM SOC REV*	10.1039/c3cs60046g	862
7	Keskin DB, 2019, *NATURE*	10.1038/s41586-018-0792-9	860
8	Brown PD, 2017, *LANCET ONCOL*	10.1016/S1470-2045(17)30441-2	769
9	Gondi V, 2014, *J CLIN ONCOL*	10.1200/JCO.2014.57.2909	756
10	Krikidis I, 2014, *IEEE COMMUN MAG*	10.1109/MCOM.2014.6957150	689

## Data Availability

The data sets analyzed in this study are available upon request from the corresponding author.

## ABBREVIATION

PPARs: Peroxisomal Proliferator-Activated Receptors
